# The Histopathology of the Diseases of the Skin

**Published:** 1896-12

**Authors:** 


**The Histopathology of the Diseases of the Skin.** By Dr. P. G. UNNA. Translated by NORMAN WALKER, M.D. Pp. xxviii., 1205. Edinburgh: William F. Clay. 1896.

After considering Anomalies of Circulation in 66 pages, nearly 600 pages of this work are taken up by inflammatory affections. Then 452 pages are given to Disturbances of Nutrition, Progressive and Regressive; 69 pages to Malformations, and 7 pages to Saprophytes and Foreign Bodies; then there is a full index, very useful, but not too complete—*e.g.* the word favus nowhere occurs. The author's preface gives us the key to the work he has accomplished. He states that, practically, since the publication of Gustav Simon's *Text-Book on Histopathology of the Skin*, in 1840, no important work on the subject has been published. The author determined to work on the same ground—viz., the whole of cutaneous pathology—and although five years of close, exact, and continuous labour (and we know what work means in Dr. Unna's laboratory) have been given to it, the author acknowledges how incomplete much of it is. Then as to "method" of work. Nuclear stains almost solely characterised the older modes, with the result that many skin affections appeared microscopically identical; but the author, by extending the process of staining to the interstitial tissues, has brought out very marked microscopical distinctions. The two pages of coloured plates near the beginning of the work illustrate these processes in several respects.

At the head of many of the divisions we have a very useful and concise clinical history of the disease under discussion, and although there are many attempts at simplification of nomenclature, this has caused the introduction of many new terms. We fear the time of simple names is yet far distant in skin affections. We owe very much to the translator; but of this we will speak at the end of our review.

We have great difficulty in summarising, because the work is very extensive and the processes of examination, in many instances, are new to us. No one is justified in criticising them until he has gone carefully and patiently through them. For example, concerning œdema of the skin it is said: "Every œdema of the lymph spaces separates the elastic fibres. Wherever we observe an elastic fibre stretched across an opening in the tissue in a different direction to the neighbouring fibres, that space was present in life, for the elastic fibres cut through in section separate from each other, when the section is pressed, in a direction always parallel to the collagenous bundles" (p. 23). Again: "The hæmorrhages of the skin are distinguished clinically as such from hyperæmia on one hand, and angioma on the other, by this fact, that their red colour does not disappear under the pressure of the diascope" (p. 44). Then the coloured plates in the beginning of the work, the results of various staining agents, illustrate our position very well.

Even when Dr. Unna theorises he gives strong grounds for his views. Speaking of the bleeding in purpura, the theory that there are poisonous foreign bodies, in particular certain bacteria, whose entry into the circulation of the skin, in some unexplained manner, and without the intervention of wide-spread thrombi, induces hemorrhage, seems reasonable, because in other affections, as erysipelas, phlegmon, and elephantiasis nostras, there are much wider-spread thromboses and yet no tendency to hemorrhage. Then we must give the author credit for conscientiousness. Thus, after describing very minutely the changes which are induced in herpes zoster on the skin-layers, and which are, in fact, well displayed in coloured plate 11, and contrasted with the ballooning of the surface in varicella as shown in plates 10 and 12, he adds: "My preparations allow of no conclusions as to changes in the nerves. To this point future investigation, with appropriate methods, must be particularly directed." (p. 154.)

Amongst the infectious inflammations, seborrhœic eczema in its many forms is, as we should have expected, dealt with with much detail and great precision. Ecthyma is regarded as a distinct disease from impetigo contagiosa; our own experience, and that of most English authors, do not corroborate this view. With regard to lichen, Dr. Unna prefers the simple term lichen planus, dropping out the adjective "ruber," which has been the cause, if not of bloodshed, yet of much confusion and strife since Hebra, sen., first employed the term. Lichen scrofulosorum (not scrophulosorum, as it is spelt p. 303, for the term has no Greek origin) is taken out of the lichen group, and is termed "Folliculitis scrophulosorum." Acne is well described, but the arrangement excludes A. varioliformis, which becomes "Folliculitis varioliformis," being differentiated from acne because the bacillus peculiar to it does not exhibit any comedo. Tricophytosis and favus come under inflammatory infectious diseases. This is certainly not illogical, but it shows the increasing importance of parasites in inflammation. We feel that we should have preferred, nevertheless, to have still found them classified under the heading of "Parasitic diseases." No confusion of frambœsia (yaws) with syphilis is entertained, and we quite agree with this view. There may be analogy in several respects, but the history is very different, and those medical men who have had most to do with yaws endorse the distinction. The subject of syphilis receives very scientific consideration. We are quite in accord with the author in making no very distinct line between secondary and so-called tertiary symptoms. He says (p. 554): "The tertiary syphilides form a sort of general group, embracing everything which can be regarded as the result of the syphilitic invasion. But nowhere is it easier to demonstrate . . . that they are only the rejuvenated remains of old syphilitic products, of the secondary, or even primary period." From pp. 624—663 we have some very interesting remarks and observations on the acute exanthemata, but, strange to say, not a word about "German measles" or "rötheln"!

In Section III., "Progressive Disturbances of Nutrition," the remarks on malignant growths form a very interesting chapter. "Paget's carcinoma of the nipple" is very fully considered, and good evidence adduced that "swelling of the lymph glands only accompanies the last stage of the general cancerous cachexia." In the succeeding chapter, "Benignant Growths," we have the name proposed by Virchow and Neisser, viz., "Epithelioma contagiosum," for "molluscum contagiosum," adopted, because "the striking elastic hardness of the nodules makes the name molluscum . . . quite inapplicable"; but we do not quite agree with this explanation, for the reason that our Latin dictionary defines the word "a kind of a soft nut, with a thin shell," which describes the state of things very fairly, and "epithelioma" has come to be associated with a more grave disorder. We also traverse the statement that Urticaria pigmentosa about the time of puberty almost always spontaneously disappears, because one of the most marked of several cases we have seen, of which we have a most excellent photograph, showed very distinct traces, although, perhaps, somewhat fading ones, at the age of 22, many years after menstruation had been set up. Unna regards the œdema of this complaint as being limited to the papillary body, and not affecting the whole skin, as in urticaria proper.

Myxœdema, which is classed under "Regressive Disturbances of Nutrition," is described as "œdema-like to the eye and not to the finger," and it shows "a wide-spread degeneration of the connective tissue framework, which alone suffices to explain the sodden, cushion-like appearances." Ulceration is said to be "granulation tissue hindered in its typical involution," a most acute observation. Alopecia areata is rightly regarded by Dr. Unna as contagious, but its micro-organism has not yet been discovered—the right staining material has not yet been found out.

Space forbids us to add more. Any review can only do scant justice to the merits of the work, but we must not overlook the translator's part. We owe a debt of deep gratitude to Dr. Norman Walker, which we cannot express better than by quoting from the author's preface to the English edition: "The attempt to make the book live again in another language is a . . . difficult task . . . But so far as I understand English, the translation has been most successful, and indeed in many places its conciseness has a certain advantage over the original." We thoroughly confirm this opinion. The text has been most ably and conscientiously rendered. That there are a few errors and a few involved, if not ambiguous, sentences, goes without saying, but these are but molehills in a mountain of intelligence—snowflakes on a summer landscape, which the warmth of a second edition will entirely dissipate. No medical library can be complete without a copy of this work; and every dermatologist and consulting physician should have the book on his shelves.

